# Temporomandibular Disorders in Brain Injury Patients and Diagnostic Accuracy of the Assessment Tools

**DOI:** 10.1111/odi.15281

**Published:** 2025-02-10

**Authors:** Mohit Kothari, Silas Alves‐Costa, Abhishek Kumar, Gustavo G. Nascimento, Jørgen Feldbæk Nielsen, Peter Svensson, Simple F. Kothari

**Affiliations:** ^1^ Hammel Neurorehabilitation Centre and University Research Clinic, Department of Clinical Medicine Aarhus University Hammel Denmark; ^2^ Department of Audiology & Speech Language Pathology, Kasturba Medical College, Manipal Academy of Higher Education Kasturba Medical College Manguluru Karnataka India; ^3^ Dentistry Graduate Program Federal University of Maranhão São Luís Brazil; ^4^ Division of Oral Diagnostics and Rehabilitation, Department of Dental Medicine Karolinska Institute Huddinge Sweden; ^5^ Academic Center for Geriatric Dentistry Stockholm Sweden; ^6^ Duke‐NUS Medical School—Oral Health Academic Program National Dental Research Institute Singapore; ^7^ National Dental Research Institute Singapore National Dental Centre Singapore Singapore; ^8^ Faculty of Dentistry National University of Singapore Singapore; ^9^ Department of Psychology and Behavioral Sciences Aarhus University Aarhus Denmark

**Keywords:** acquired brain injury, diagnostic validity, neurorehabilitation, stroke, temporomandibular disorders

## Abstract

**Objectives:**

To evaluate the presence, severity and progression of temporomandibular disorders (TMD) in acquired brain injury (ABI) population and determine the diagnostic accuracy of 3Q/TMD and Fonseca Anamnestic Index (FAI) against the gold standard, Diagnostic Criteria for TMD (DC/TMD).

**Methods:**

ABI individuals were assessed using 3Q/TMD and FAI at admission (*n* = 73) and Week 4 (*n* = 52), while DC/TMD was conducted only at Week 4. Diagnostic accuracy, sensitivity and specificity of 3Q/TMD and FAI were calculated against DC/TMD.

**Results:**

TMD was detected in 66.0% (3Q/TMD) and 27.8% (FAI) at admission, with mild (75%) to moderate (25%) severity which was mostly pain‐related. TMD frequency decreased to 11.3% (3Q/TMD) and 17.3% (FAI) by Week 4. Accuracy rates were 0.82 for 3Q/TMD and 0.83 for FAI. Sensitivity was 0.33 (3Q/TMD) and 0.50 (FAI), while specificity was high at 0.93 (3Q/TMD) and 0.90 (FAI).

**Conclusions:**

TMD presence was higher in an ABI population compared to the general population however the frequency decreased over time. The high specificity and accuracy of 3Q/TMD and FAI indicate their potentials as screening tools but their low sensitivity limits their effectiveness in identifying all TMD cases. Patients with ABI should be assessed for TMD as part of their comprehensive care.

## 1 Introduction

Acquired brain injury (ABI), which includes stroke and traumatic brain injury (TBI), is a major health concern and the main cause of disability and death in the high‐income countries (Marshall et al. 2015; Sarti et al. 2000; Sussman et al. 2018; Wolfe 2000). In Denmark, over 230,000 people live with ABI (Tibæk et al. 2018) with a prevalence of 4.4% of TBI and 2.1% of stroke in Danish population (Vestergaard et al. 2020). Depending on the severity of the central nervous system damage, sensorimotor deficits, speech and swallowing impairments, and psychological symptoms accompany stroke and TBI (Mayer and Esquenazi 2003; Sinanović 2010). Post‐ABI sensorimotor deficits in the stomatognathic system that include the masticatory muscles, temporomandibular joint (TMJ), ligaments and related salivary glands, may result in decreased bite force, reduced lip and tongue strength, and impaired masticatory performance (Mayer and Esquenazi 2003; Sharma et al. 2019; Wolfe 2000; You et al. 2018). Furthermore, TBI is often shown to be associated with facial traumas including jaw injury, further affecting the masticatory muscles and surrounding structures that may lead to temporomandibular disorders (TMDs) (You et al. 2018).

TMDs, a heterogenous group of musculoskeletal conditions, are clinically characterized by signs and symptoms such as limitation of jaw movements, pain in the TMJ or masticatory muscles, joint noises and jaw locking (Cadden 2009; Schiffman et al. 2014; Scrivani, Keith, and Kaban 2008). TMD is the most common cause of chronic pain of non‐dental origin in the orofacial area, affecting around 5%–12% of the general population, primarily affecting females and individuals within the 20–40 age range (Lövgren et al. 2016a). The multifactorial etiology of TMD is consistent with a biopsychosocial model of illness, and up to 76% of people with TMDs have moderate‐to‐severe somatization, while 60% have depression (De La Torre Canales et al. 2018; Slade et al. 2013). Despite the pathophysiology of TMD being based on various interrelated factors, changes in the neuromuscular system occur, which is reflected by dysfunction and functional limitations of natural jaw and oral tasks such as eating, swallowing, communicating, and freely moving the lower jaw (Anderson et al. 2010). Current evidence indicate that stroke and TBI, both central nervous system lesions, can lead to compromised orofacial functions, potentially causing and even aggravating TMDs (Kemppainen et al. 1999; Schimmel et al. 2017). Studies on this topic in individuals with ABI are mostly focused on the assessment of quality of mastication, lip, and bite force measurements, which are indirectly related to TMD assessment. Thus, further research is needed that can evaluate the presence, progression, and severity of TMD directly in patients with ABI.

Currently, several protocols are available in the literature for the diagnosis and classification of TMD, such as the Helkimo Index, American Academy of Orofacial Pain (AAOP) Temporomandibular Disorder Questionnaire, Fonseca Anamnestic Index (FAI), Research Diagnostic Criteria for Temporomandibular Disorders (RDC/TMD), and the current Diagnostic Criteria for Temporomandibular Disorders (DC/TMD) (Anderson et al. 2010; de Santis et al. 2014; Schiffman et al. 2014; Stasiak et al. 2023; Zhao et al. 2011). Some of these instruments such as DC/TMD (Schiffman et al. 2014), a gold standard for diagnosing TMD, are rather complex and time‐consuming, involving face‐to‐face interviews and requires calibrated/trained personnel to perform clinical examinations, whereas others, such as self‐reported questionnaires, despite being faster and simpler, may have lower diagnostic accuracy (Sánchez‐Torrelo et al. 2020). While there are tools that are quite robust in diagnosing TMD in general, they may not be tailored to the unique needs of individuals with ABI, and none of them has been specifically developed or tested for individuals with ABI. Developing tailored assessment tools and refining diagnostic methods for this population would enhance the understanding of TMD and may improve the patient care for TMD in individuals with ABI.

Contemporary TMD screening tools that are robust in the identification of TMDs include, the 3Q/TMD, and the FAI (Lövgren et al. 2016b; Stasiak et al. 2023). To the best of our knowledge no study has so far directly evaluated the relationship between TMD and ABI. From this point of view, our study aimed to evaluate the presence, severity, and progression of TMD in patients with ABI admitted at a neurorehabilitation center. The secondary aim was to determine the diagnostic accuracy and validity of the 3Q/TMD and FAI in comparison to the gold standard, DC/TMD in individuals with ABI. The null hypothesis was that the 3Q/TMD and FAI would be less accurate when compared with the DC/TMD.

## 2 Material and Methods

### 2.1 Study Design, Participants, and Recruitment

All individuals with ABI aged ≥ 18 years admitted at the Hammel Neurorehabilitation Center and University Research Clinic (HNRC), were recruited in a longitudinal observational cohort study. In the current study, ABI was defined as a brain injury resulting either from trauma (head injury, physical trauma due to accident and assault, neurosurgery, etc.) or non‐traumatic injury derived from either an internal or external source like stroke, brain tumor, infection, hypoxia, and anoxia.

**FIGURE 1:**
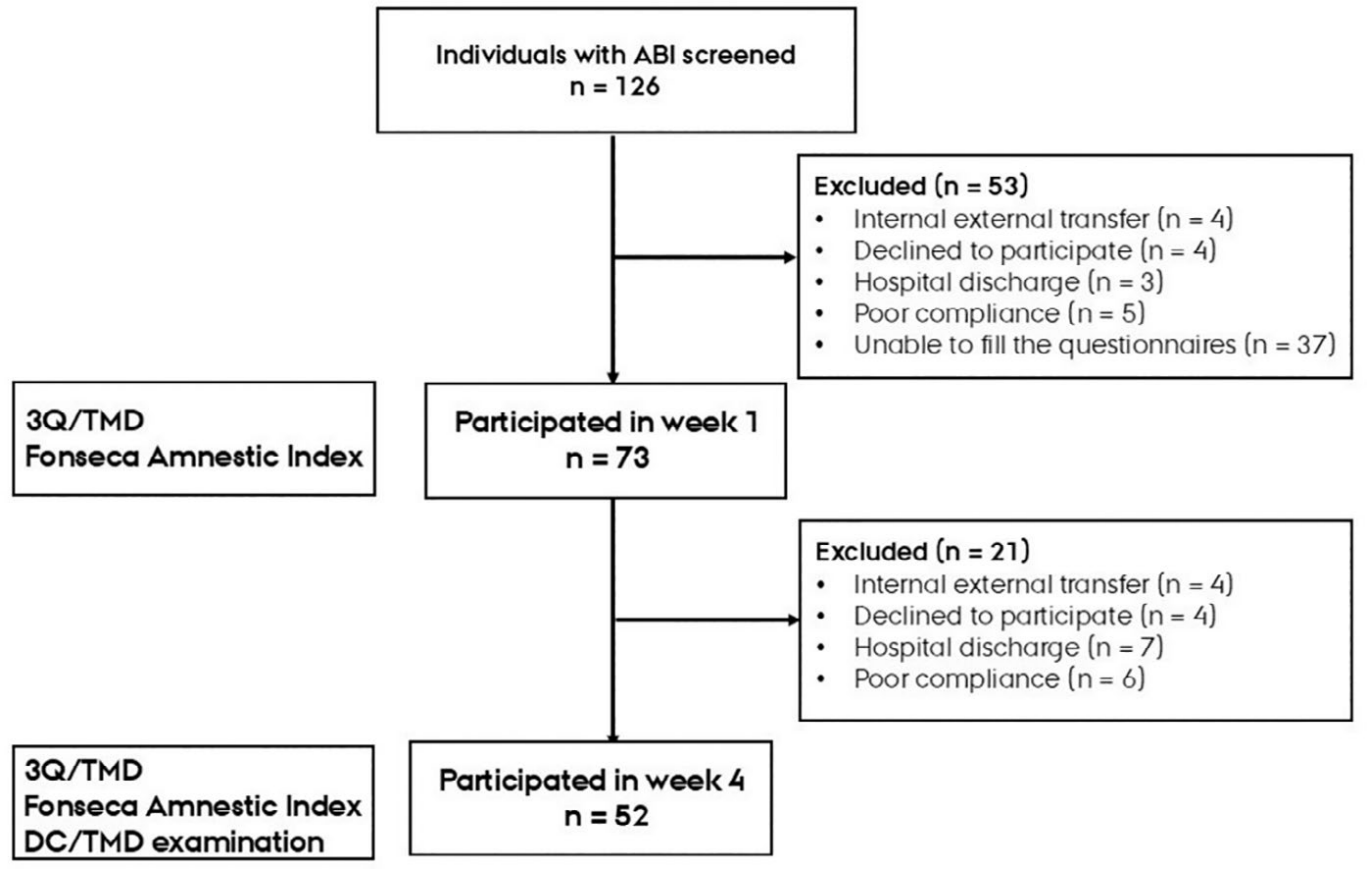
Flowchart. ABI, acquired brain injury; DC/TMD, Diagnostic Criteria for TMD; TMD, temporomandibular disorders.

A total of 126 hospitalized individuals with ABI were initially screened and examined within the first Week of admission (Week 1) to assess the presence and severity of TMD. At Week 4, these individuals were re‐examined to evaluate the progression of TMD. Of the 126 individuals, 73 met the eligibility criteria for the Week 1 assessment, and 52 were eligible for the follow‐up examination at Week 4 after fulfilling all the eligibility criteria (Figure 1).

Individuals admitted for any other reasons than ABI, such as neurodegenerative disorders and polyneuropathy, were excluded. Pregnant women were also excluded, as hormonal changes that occur during pregnancy might influence the oral and orofacial health assessment (LeResche et al. 2005). Some patients moved in and out of HNRC due to medical emergencies or owing to the need for other facilities that were unavailable at HNRC. In case those patients were re‐admitted within 5 days to the HNRC from their first day of admission, they were included in the study The readmission day was counted as their first day of admission. If the individuals with ABI had difficulties such as fatigue/cognition/limited mouth opening/stress/infection, that did not allow the examination, they were rescheduled within 1 week and were excluded from the study if it was not possible to re‐examine within a one‐week window (Figure 1) (Kothari et al. 2020). The project was notified to the Central Denmark Region Committee on Health Research Ethics with case number: 1–10–72‐124‐22. According to the Danish Consolidation Act on Research Ethics Review of Health Research Projects with Consolidation Act number 1338 of 1 September 2020 section 14 (1), the committee did not consider the current project as a health research project (section 2 (1)) and concluded it as a “clinical quality development project”. Hence, no ethical approval and no written informed consent is required from the participants. However, the permission of the institutional review board for clinical project was taken under case number: 196525. This study is performed in accordance with the Helsinki Declaration II and participants were informed about their participation through verbal and written project information and their free will to decline at any time of the project.

## 3 Procedures and Measures

### 3.1 Demographics and Socio‐Behavioral History

After the inclusion of individuals with ABI, a self‐administered structured questionnaire was used to elicit social and behavioral history in relation to oral and orofacial health. The questionnaire collects information on age, gender, education level (basic: 1–9 years, vocational: technical degree; high school: 9–12 years; higher education: university degree), smoking habits, and dental appointment frequencies (prior to hospitalization) among others. The full list of variables collected is available in Table 1.

**TABLE 1:** Characteristics and demographics.

Characteristics of participants	Week 1 (*n* = 73)	Week 4 (*n* = 52)
Age, mean years (SD)	55.28 (15.1)	54.98 (15.4)
Sex, *N* (%)		
Male	52 (72.2)	39 (75.0)
Female	20 (27.8)	13 (25.0)
Body mass index, mean (SD)	26.31 (4.4)	26.43 (4.2)
Primary diagnosis, *N* (%)		
Stroke	58 (80.6)	44 (84.6)
TBI	10 (13.9)	5 (9.6)
Others	4 (5.6)	3 (5.8)
Onset of brain injury, mean days (SD)	34.54 (30.0)	
Stay in acute care, mean days (SD)	16.39 (14.2)	
Dysphagia, *N* (%)		
Present	20 (27.8)	9 (17.3)
Absent	52 (72.2)	43 (82.7)
Eating difficulties, *N* (%)		
Present	10 (15.6)	3 (6.0)
Absent	54 (84.4)	47 (94.0)
Feeding status, *N* (%)		
Oral	61 (84.7)	48 (92.3)
Tube fed	11 (15.3)	4 (7.7)
Pneumonia, *N* (%)		
Present	19 (26.4)	12 (23.1)
Absent	53 (73.6)	40 (76.9)
Diabetes, *N* (%)		
Present	13 (18.1)	6 (11.5)
Absent	59 (81.9)	46 (88.5)
Hypertension, *N* (%)		
Present	41 (56.9)	30 (57.7)
Absent	31 (43.1)	22 (42.3)
Smoking, *N* (%)		
Never	30 (41.7)	
Former	32 (44.4)	
Current	10 (13.9)	
Dental visit in the last 12 months, *N* (%)		
Not once	17 (24.3)	
Once	25 (35.7)	
Twice	21 (30.0)	
Three times	3 (4.3)	
Four times	4 (5.7)	

Abbreviation: SD, standard deviation.

### 3.2 Medical Records

Patients' primary diagnosis, medical history (diabetes, hypertension etc.), the onset of brain injury, length of stay in acute care were documented at baseline and at Week 4 from patients' medical records (Table 1). As part of the routine clinical practice at HNRC, dysphagia, eating difficulties, feeding status, onset of pneumonia, body mass index, and other relevant brain injury related scores are clinically assessed at every fourth week by certified health professionals to monitor the progress and rehabilitation needs of the patients. These scores were documented at baseline and Week 4 from patients' medical records (Table 1).

### 3.3 Assessment of Temporomandibular Disorders (TMD)

#### 3.3.1 3Q/TMD Questionnaire

This is a simple, cost‐effective, and validated tool for screening TMD (Lövgren et al. 2016b). It consists of three questions, in which two questions addresses frequent pain in the temporomandibular region indicative of pain related TMD, and one question addresses frequent locking and catching of the TMJ indicative of intraarticular TMD. The three screening questions were as follows:

Q1: “Do you have pain in your temple, face, jaw, or jaw joint once a week or more?,” Q2: “Do you have pain once a week or more when you open your mouth or chew?” Q3: “Does your jaw lock or become stuck once a week or more?”

It was used at admission (Week 1) and at follow‐up (Week 4). Individuals with an affirmative answer to at least one of the 3Q/TMD are classified as 3Q‐positives, whereas individuals with negative answers to all three questions are classified as 3Q‐negatives.

#### 3.3.2 Fonseca Anamnestic Index (FAI)

This is a reliable and validated questionnaire, which allows diagnosis, presence, and classification of TMD according to the severity (Fonseca et al. 1994; Stasiak et al. 2023). It was used at admission (Week 1) and at follow‐up (Week 4). It is composed of 10 questions with three response options (0 = no; 5 = sometimes; and 10 = yes). The overall score ranges from 0 to 100. The patients are classified according to their total score, where 0–15 means no TMD, those with a score of 20–40 have mild TMD, those with a score of 45–60 have moderate TMD, and those between 70 and 100 have severe TMD.

The following 10 questions compose the instrument: (Q1) “Do you have difficulty opening your mouth wide?”, (Q2) “Do you have difficulty moving your jaw to the sides?”, (Q3) “Do you feel fatigue or muscle pain when you chew?”, (Q4) “Do you have frequent headaches?”, (Q5) “Do you have neck pain or a stiff neck?”, (Q6) “Do you have ear aches or pain in that area (temporomandibular joint)?”, (Q7) “Have you ever noticed any noise in your temporomandibular joint while chewing or opening your mouth?”, (Q8) “Do you have any habits such as clenching or grinding your teeth?”, (Q9) “Do you feel that your teeth do not come together well?”, (Q10) “Do you consider yourself a tense (nervous) person?” (Fonseca et al. 1994; Stasiak et al. 2023).

#### 3.3.3 Diagnostic Criteria for Temporomandibular Disorders (DC/TMD)

The DC/TMD demonstrates a high level of precision and reliability in the diagnosis of muscular TMD, and its use has become widespread as a standard diagnostic tool for TMD, being considered the gold standard (Schiffman et al. 2014). The DC/TMD protocol consists of a Symptom Questionnaire followed by a physical examination. This instrument allows for the identification of patients with a range of simple to complex TMD presentations and differentiates between painful and/or non‐painful types of TMD. It is appropriate for use in both clinical and research settings (Schiffman et al. 2014).

The 3Q/TMD, FAI and symptom questionnaire from the DC/TMD were filled out by the project nurse after the patients answered the questions from the questionnaires verbally, while the DC/TMD clinical examination was performed by a trained dentist having DC/TMD certification.

We performed a feasibility study (*n* = 5) prior to the main study, where we assessed TMD using 3Q/TMD, FAI and the DC/TMD. In this pilot study, it became evident that the comprehensive DC/TMD clinical examination could not be successfully performed and completed in ABI patients during their first week of admission. This was because individuals with ABI at admission are prioritized with more complex rehabilitation plans as they have severe functional, cognitive, and motor disabilities. Given the practical challenge with patients' compliance, time constraints and DC/TMD intricate algorithms, the DC/TMD examination was therefore only conducted at Week 4.

### 3.4 Analytical Approach

Descriptive statistics for the variables were performed, where the mean and its respective standard deviation were obtained for continuous variables. At the same time, the absolute and relative frequencies were reported for categorical variables at baseline and Week‐4 assessments. The presence of TMD was measured using all three (3Q/TMD, FAI, and DC/TMD) instruments, while the severity of TMD was assessed using FAI.

Diagnostic accuracy metrics, including sensitivity, specificity, receiver operating characteristic (ROC) and area under the curve (AUC), were calculated to compare the performance of the 3Q/TMD and FAI instruments against the reference gold standard, DC/TMD at the Week‐4 assessment. Positive and negative predictive values (PPV and NPV) were also calculated. The AUC was used to summarize the overall diagnostic ability of the tests, with values closer to 1 indicating better discrimination. The optimal diagnostic threshold was determined using Youden's index, which simultaneously maximizes sensitivity and specificity. Confidence intervals for the AUC and the threshold were obtained using bootstrap methods for robust estimation. All analyses were performed in R version 4.3.3.

## 4 Results

### 4.1 Characteristics and Demographic Findings

The characteristics and demographic findings of participants at baseline and Week 4 are presented in Table 1. The mean age of participants was around 55.2 years (±15.2 years). The majority of the participants were men (> 70%), and most of the participants had stroke as their primary diagnosis (81%), followed by TBI (14%) at admission. The mean time since the onset of brain injury was 35 days (±30) at baseline assessment (Week 1). The average length of stay in acute care was 17 days (±14) before they were admitted to the neurorehabilitation center (HNRC).

There was a noticeable decrease in the number of participants diagnosed with pneumonia and diabetes from Week 1 to Week 4. The proportion of participants with hypertension did not change throughout the entire study period of 4 weeks. There was also a substantial decline in the percentage of participants reporting dysphagia and eating difficulties during the time frame (Table 1). Moreover, the proportion of participants feeding orally increased while those being tube‐fed decreased. Approximately 24% of participants reported no dental visits in the past 1 year before admission to HNRC.

### 4.2 Presence, Severity, and Progression of TMD Through Different Instruments

#### 4.2.1 3Q/TMD

At Week 1, 66.0% (*n* = 37) of the participants were diagnosed with TMD and by Week 4, this number dropped to 11.3%, showing a percentage reduction of 82.9% over the study period. All the patients diagnosed with TMD at both Week 1 and Week 4, reported pain‐related TMD (Table 2). Additionally, 89.2% (*n* = 33) of the TMD patients at baseline were diagnosed with intra‐articular TMD, which decreased to 16.6% (*n* = 1) in 4 weeks (Table 2).

**TABLE 2:** Presence, severity, and course of TMD through different instruments.

TMD instruments	Week 1 N (%)	Week 4 N (%)
3Q/TMD	***n* = 56**	***n* = 52**
Absent	19 (33.9)	46 (88.7)
Present	**37 (66.1)**	**6 (11.3)**
Diagnosis		
Pain related TMD	37 (100.0)	6 (100.0)
Intra‐articular TMD	33 (89.2)	1 (16.6)
Fonseca Anamnestic Index	***n* = 73**	***n* = 52**
Absent	53 (72.6)	43 (82.7)
Present	**20 (27.8)**	**9 (17.3)**
Severity		
Mild	15 (75.0)	8 (88.8)
Moderate	5 (25.0)	1 (11.1)
Severe	0 (0.0)	0 (0.0)
DC/TMD		
Absent	—	44 (84.6)
Present	—	**8 (15.4)**
Diagnosis		
Pain‐related TMD		4 (50.0)
Intra‐articular TMD		5^a^ (62.5)

*Note:* Bold indicates a number of participants screened (n) and subsequently diagnosed with TMDs using different instruments.

Abbreviations: DC/TMD, diagnostic criteria for TMD; TMD, temporomandibular disorders.

^a^ Indicates one patient had diagnoses of both pain‐related and intra‐articular TMD.

#### 4.2.2 Fonseca Anamnestic Index (FAI)

At Week 1, 27.8% (*n* = 20) of participants were diagnosed with TMD, with 75.0% (*n* = 15) having mild TMD and 25.0% having moderate TMD according to the FAI criteria (Table 2). By Week 4, the overall diagnosis dropped to 17.3% (*n* = 9), with only 11.0% having moderate TMD reported and 88.8% having mild TMD (Table 2). The chance of having TMD decreased over the study period with a percentage reduction of 37.8%. In addition, 55.0% of all patients diagnosed with TMD, reported frequent headaches and 45.0% neck pain; 55.0% considered themselves as tensed/nervous people; and 65.0% reported to clench or grind their teeth according to the FAI at Week 1.

#### 4.2.3 Diagnostic Criteria for Temporomandibular Disorders (DC/TMD)

DC/TMD showed that 15.4% (*n* = 8/52) of participants had a positive TMD diagnosis at Week 4. Of these, 50.0% (*n* = 4) had pain‐related TMD, two patients with local myalgia, one with myofascial pain and one with arthralgia. Further, 62.5% (*n* = 5) had intra‐articular TMD—three with disc displacement (DD) with reduction, one with DD without reduction without limited opening and one with DD with reduction and degenerative joint disease (Table 2). The average pain‐free, maximum unassisted, and maximum assisted mouth opening in participants with positive TMD diagnosis was 39.3 ± 7 mm, 42.9 ± 8 mm, and 46.5 ± 10 mm respectively. The average protrusion, right lateral and left lateral deviation was 4.0 ± 2.9 mm, 6.9 ± 3.1 mm, and 7.9 ± 2.7 mm respectively.

### 4.3 Diagnostic Validity and Accuracy of 3Q/TMD and FAI to DC/TMD

**FIGURE 2:**
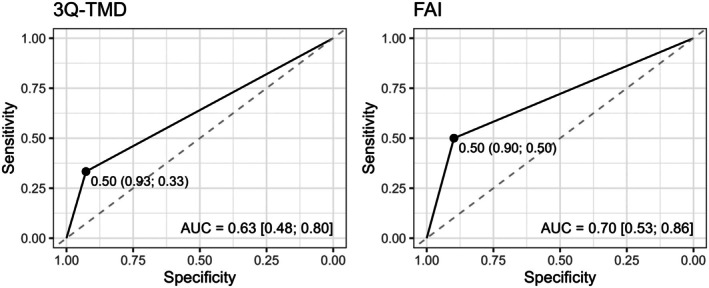
Diagnostic validity and accuracy of 3Q/TMD and FAI to DC/TMD. AUC, area under the curve; DC/TMD, Diagnostic Criteria for TMD; FAI, Fonseca Anamnestic Index; TMD, temporomandibular disorders.

The 3Q/TMD and FAI demonstrated accuracy rates of 0.82 (AUC: 0.63; 95% CI: 0.48; 0.80) and 0.83 (AUC: 0.70; 95% CI: 0.54; 0.86) respectively, for identifying any type of TMD, that is both painful and non‐painful subtypes (Table 3). Sensitivity for the 3Q/TMD was 0.33 (95% CI: 0.11; 0.67), and for the FAI, it was 0.50 (95% CI: 0.20; 0.80). Specificity was high for both tools, with 0.93 (95% CI: 0.83; 1.00) for the 3Q/TMD and 0.90 (95% CI: 0.82; 0.98) for the FAI (Figure 2). Both tools showed positive predictive values (PPV) with the 3Q/TMD reporting a PPV of 0.50 (95% CI: 0.12; 1.00) and the FAI reporting 0.50 (95% CI: 0.25; 0.80). Negative predictive values (NPV) were notably higher, with the 3Q/TMD having an NPV of 0.86 (95% CI: 0.81; 0.93) and the FAI having 0.90 (95% CI: 0.85; 0.96).

**TABLE 3:** Diagnostic validity and accuracy of 3Q/TMD and FAI to DC/TMD.

TMD instrument	Specificity (95% CI)	Sensitivity (95% CI)	Accuracy (95% CI)	NPV (95% CI)	PPV (95% CI)
3Q/TMD	0.93 (0.83–1.00)	0.33 (0.11–0.67)	0.82 (0.74–0.90)	0.86 (0.81–0.93)	0.50 (0.12–1.00)
Fonseca	0.90 (0.82–0.98)	0.50 (0.20–0.80)	0.83 (0.75—0.92)	0.90 (0.85–0.96)	0.50 (0.25–0.80)

Abbreviations: NPV, negative predictive values; PPV, positive predictive values.

## 5 Discussion

The present study demonstrated that the presence of TMD was as high as up to 66% (*n* = 37/56) in individuals with ABI admitted at a neurorehabilitation center, which appears to be exponentially higher compared to the general population (5%–12%) when using the same diagnostic tool, that is, the 3Q/TMD (Lövgren et al. 2016a). A recent meta‐analysis that considered factors such as geographical region, patient age, and sample size, reported a global TMD prevalence of approximately 31% in adults, mostly affecting the age range of 18–60 years (Zieliński, Pająk‐Zielińska, and Ginszt 2024). Another meta‐analysis confirmed a similar TMD prevalence of 31% using the gold standard, RDC/TMD or DC/TMD examination (Valesan et al. 2021). These meta‐analyses further indicate a higher prevalence of TMD in the ABI population compared to the general population. The sheer number of TMDs in the general population, as well as in the current study in individuals with ABI, highlights the significance of this condition which can impose a substantial economic and social burden.

Head and neck injuries are well established risk factors for TMD with up to 25% of TMD patients being linked to the history of trauma (Chisnoiu et al. 2015; Huang et al. 2002; Svensson and Kumar 2016). TBI is shown to be associated with facial traumas including jaw injury further affecting the masticatory muscles and structures surrounding them and may lead to TMDs such as, myofascial pain and jaw joint pain (arthralgia) (Huang et al. 2002; Sharma et al. 2019; You et al. 2018). A recent study by Karpuz et al. found that the frequency of TMD using FAI was 80% in patients with TBI (Karpuz, Yılmaz, and Yılmaz 2023). In terms of mechanisms it can be speculated that after brain injury, microglia become and may remain activated, which leads to an exaggerated neuroinflammatory response defined as central sensitization, a potential pathway that could explain the relationship between brain injury and TMD (Ji et al. 2018; Just and Schwulst 2021). This state of increased reactivity lowers the threshold for painful stimuli and is associated with the development and maintenance of chronic pain, which is a key mechanism for both neuropathic pain and inflammatory pain, a process implicated in the development of both TBI and TMD (Ji et al. 2018; Just and Schwulst 2021; Kothari et al. 2016, 2015). In a stroke population, approximately 50% of patients with hemiparesis are known to have facial control deficiency and weakened orofacial and mandibular functions, potentially contributing to the development of TMD (Ramos et al. 2019). Studies have shown that the neurological diseases may disrupt TMJ biomechanics and reduce the mandibular range of motion, especially in cases of facial paralysis, leading to muscular, and postural imbalances (Oh, Kang, and Kim 2013; Ramos et al. 2019; Schimmel et al. 2017; Valesan et al. 2021). Postural and movement defects in the craniocervical region and impairments in their control may be the main reasons for the decline in stomatognathic system function, including the TMJ, muscles, and the onset of swallowing problems (Oh, Kang, and Kim 2013).

In the current study, all the individuals diagnosed with TMD in accordance with 3Q/TMD reported painful TMD both at admission and at Week 4. In addition, according to DC/TMD, 50% of patients with positive TMD diagnosis had pain‐related TMD, with the majority having muscular pain origin that could be attributed to central sensitization as mentioned above. It has also been reported that primary clinical factor associated with TMD pain onset is the presence of other pain conditions (Maixner, Diatchenko, and Dubner 2011). Concurrent episodes of headache and other body pain are shown to be strongly associated with TMD symptoms including painful TMD (Slade et al. 2013). Recently, a study showed that mild TBI patients with painful TMD reported significantly higher headache intensity and increased symptom burden compared to the patients without painful TMD (Kothari et al. 2024). In the current study, ⁓50.0% of patients with the presence of TMD reported to have headaches and/or neck pain. TMD and headache are comorbid conditions with significant overlap between them (Tchivileva et al. 2017). Given that the headache and TMD are chronic pain conditions mediated by the same nerve (trigeminal) and share anatomical locations, it can be speculated that the presence of central changes in one of these disorders could facilitate the incidence of the other (Herrero Babiloni et al. 2020). Furthermore, it could be related to the risk factors that are attributed to the onset and persistence of TMD such as psychological distress (frequency of somatic symptoms, anxiety and depression), pain amplification (pro‐inflammatory states, impaired pain regulation, autonomic function, and neuroendocrine function), or genetic factors which need to be addressed further in this population (Kothari, Baad‐Hansen, and Svensson 2017; Maixner, Diatchenko, and Dubner 2011; Mitrirattanakul and Merrill 2006; Slade 1997). The majority of the TMD positive patients diagnosed in accordance with the DC/TMD also had intra‐articular TMD, with DD with reduction being more frequent compared to other intra‐articular TMD. DD with reduction is also the most prevalent (25.9%) TMJ disorder among general population (Valesan et al. 2021). One of the strong causative factors of TMD with DD is macrotrauma, such as traffic accidents, falls, and assaults (Yun and Kim 2005). Any traumatic event involving TMJ may modify the preexisting relationship of the TMJ and related structures, potentially leading to multiple problems, such as joint effusion, dislocation, disc displacement, and restricted jaw movement (Lee, Lee, and Auh 2021; Yun and Kim 2005).

On another note, we observed that after 4 weeks of hospitalization, the TMD reduced substantially by 82.9% and 37.8%, using the 3Q/TMD and FAI respectively, in the ABI population. It has been commonly observed that the rationale behind the recovery‐related process, particularly spontaneous recovery is strongly time‐dependent in patients with ABI (Grefkes and Fink 2020). Following the onset of brain injury, the brain undergoes quick functional reorganization, especially in the first 24 h (hyperacute phase), the first 7 days (acute phase) and the first 3 months (early sub‐acute phase) (Grefkes and Fink 2020; Kwakkel et al. 2003). The ABI population in our study aligns with a similar onset period (early sub‐acute phase), supporting the notion for the observed decline in TMD over the 4 weeks of hospitalization (Table 1). Already within hours after the onset of brain injury (cerebral ischemia), a cascade of plasticity‐enhancing mechanisms leads to dendritic growth, axonal sprouting, and the formation of new synapses (Kitagawa 2007). The “proportional recovery rule” is an interesting concept that assumes recovery of function follows a fundamental neurobiological process, and patients, on average improve around 70% (± 15%) of their lost function within 3–6 months after stroke (Stinear 2017). Neuroimaging studies, including functional magnetic resonance imaging, also confirms the neural mechanisms about brain reorganization after stroke, revealing the recovery of function (Grefkes and Fink 2020). Even though we observed a decline in TMD cases after 4 weeks of hospitalization, it was clear that TMD was still higher in the ABI population compared to the general population (Lövgren et al. 2016a). Thus, it is important to evaluate and manage TMD in ABI patients as it can have significant impact on jaw functions such as eating, swallowing, communication etc. and may complicate the rehabilitation plan and recovery.

The screening tools used in the current study are simple and easy to use. Yet, the results should be interpreted with caution as the mentioned screening tools have never been validated in the ABI population. Hence, it was imperative to test the validity of the screening tools, which can help in better planning and providing appropriate intervention to minimize TMD chronicity in the ABI population. Therefore, DC/TMD, a standard diagnostic tool for TMD (Schiffman et al. 2014) was employed, and the results revealed that the screening tools tested, had high specificity and accuracy but lacked adequate sensitive, indicating that they may miss a substantial portion of the true positive cases of TMD, particularly those with milder symptoms or atypical presentations. Screening tools like 3Q/TMD and FAI appear not sufficiently sensitive to diagnose TMD in ABI patients but on the other hand, the standard diagnostic method, DC/TMD seems to be an ideal tool; however, its use may be limited, given its time‐consuming assessment protocol. Thus, it may not be suitable for patients with acute stage ABI who have significant functional, cognitive, and motor issues. Moreover, DC/TMD involves a thorough physical examination that looks at several aspects of jaw function and pain provocation, a complex algorithm to reach to the final diagnosis, and the examiner must be sufficiently trained to perform the examination (Schiffman et al. 2014). Therefore, in the current situation, it is a challenge either to pick the validated tool or easy and fast‐to‐use screening tools. Even though 3Q/TMD and FAI lacked sensitivity, they both achieved a high specificity and accuracy of approximately 80%. The 3Q/TMD, comprising three questions (3Q) which attempts to separate non‐painful/painful TMDs, can be a potential TMD screening tool in the hospital settings, especially in ABI population as it does not require special training and can be performed by any health professionals. On the other hand, FAI used in the current study do not differentiate individuals into painful and non‐painful TMDs, instead focuses solely on severity, which could limit its diagnostic utility. Furthermore, TMD pain screeners that target various aspects of orofacial pain was not used in the current study as it can only provide information on painful TMD (Gonzalez et al. 2011). However, this study aimed to assess the presence of both painful and non‐painful TMD. Nevertheless, it is important that after assessing the patients with screening tools, a more comprehensive DC/TMD examination should be considered and performed whenever appropriate or at least before the patients discharge from the hospital. Such recommendations should be made together with the oral health professionals in such centers.

Overall, TMDs are a significant public health problem recognized to have a considerable impact on quality of life (Durham, Newton‐John, and Zakrzewska 2015) owing to increased healthcare costs, disability, and loss of productivity (Dworkin and LeResche 1993; Maixner, Diatchenko, and Dubner 2011). The high presence of TMD in ABI patients may compromise their rehabilitation plan, hamper the recovery and complicate the management. It is, therefore, pivotal that the health professionals should consider evaluation of TMD signs and symptoms in ABI patients during their hospitalization and the oral health professions should enquire about the ABI and direct the treatment accordingly.

### 5.1 Study Limitations

The relatively small sample size of our study limits us from further exploring the correlation between 3Q/TMD pain versus DC/TMD pain, and 3Q/TMD non‐pain versus DC/TMD non‐pain related conditions at the risk of misleading results due to an underpowered sample. However, our analyses shed light on alternative and simple methods for identifying TMDs in individuals with ABI. Future projects should disentangle the performance of these instruments. Another limitation was the cross‐sectional nature of the study, which prevents us from establishing any causal relationships. Furthermore, use of self‐reported questionnaires might have introduced recall bias.

## 6 Conclusion

TMD was present in 66% and 28% of ABI patients according to 3Q/TMD and FAI respectively at baseline and was frequently pain‐related and of mild degree. The presence of TMD appears to be higher in hospitalized ABI patients compared to the general population (5%–12%). However, the frequency of ABI patients having TMD decreased over the hospitalization time.

Both the 3Q/TMD and FAI demonstrated moderate validity, with high specificity and overall accuracy indicating that 3Q/TMD and FAI may serve as a suitable initial screening tool for assessing TMD in patients with ABI. Nonetheless, the discrepancy between 3Q/TMD and FAI at baseline (66% and 28%, respectively), their low positive predictive value and sensitivity, warrants cautious interpretation of their clinical significance. To ensure accurate diagnosis and effective TMD management, these tools should ideally be supplemented with a comprehensive DC/TMD examination in consultation and collaboration with oral health specialists. The finding of the present study, shedding light on the high presence of TMD indicates that future research is necessary in this area in ABI patients. Moreover, health professionals should focus on these disorders in ABI patients as it may increase their disability, hamper their rehabilitation plan, and negatively affect their quality of life.

## Author Contributions

**Mohit Kothari:** conceptualization, methodology, supervision, resources, writing – review and editing, writing – original draft, project administration, investigation, funding acquisition. **Silas Alves‐Costa:** formal analysis, validation, writing – review and editing, software, data curation. **Abhishek Kumar:** conceptualization, resources, writing – review and editing, methodology. **Gustavo G. Nascimento:** conceptualization, data curation, methodology, writing – review and editing. **Jørgen Feldbæk Nielsen:** project administration, writing – review and editing, visualization. **Peter Svensson:** supervision, validation, writing – review and editing. **Simple F. Kothari:** conceptualization, methodology, data curation, investigation, validation, writing – original draft, writing – review and editing.

## Conflicts of Interest

The authors declare that they have no known competing financial interests or personal relationships that could have appeared to influence the work reported in this paper.

## Data Availability

The data that support the findings of this study are available on request from the corresponding author. The data are not publicly available due to privacy or ethical restrictions.
